# Exercise increases sphingoid base-1-phosphate levels in human blood and skeletal muscle in a time- and intensity-dependent manner

**DOI:** 10.1007/s00421-014-3080-x

**Published:** 2014-12-18

**Authors:** Marcin Baranowski, Agnieszka U. Błachnio-Zabielska, Małgorzata Charmas, Jørn W. Helge, Flemming Dela, Monika Książek, Barbara Długołęcka, Andrzej Klusiewicz, Adrian Chabowski, Jan Górski

**Affiliations:** 1 Department of Physiology, Medical University of Białystok, Mickiewicza 2c, 15-222 Białystok, Poland; 2 Department of Biochemistry and Physiology, Faculty of Physical Education and Sport in Biała Podlaska, Józef Piłsudski University of Physical Education in Warsaw, Biała Podlaska, Poland; 3 Department of Biomedical Sciences, Xlab, Center of Healthy Aging, University of Copenhagen, Copenhagen, Denmark

**Keywords:** Ceramides, Dihydrosphingosine, Lipids, Red blood cells, Sphingolipids, Thrombocytes

## Abstract

**Purpose:**

Sphingosine-1-phosphate (S1P) regulates cardiovascular function and plays an important role in muscle biology. We have previously reported that cycling exercise increased plasma S1P. Here, we investigated the effect of exercise duration and intensity on plasma and skeletal muscle S1P levels.

**Methods:**

In the first experiment, 13 male athletes performed a 60-min exercise at 65 % of *V*O_2max_ and a graded exercise until exhaustion on a rowing ergometer. Samples of the venous blood were taken, and plasma, erythrocytes and platelets were isolated. In the second experiment, ten male moderately active subjects performed three consecutive periods of one-leg knee extension exercise (at 25, 55 and 85 % of the maximal workload). Muscle biopsies and blood samples from the radial artery and femoral veins were taken.

**Results:**

Under basal conditions, S1P was released from the leg, as its concentration was lower in the arterial than in the venous plasma (*p* < 0.01). Exercise until exhaustion increased plasma S1P and sphinganine-1-phosphate (SA1P) concentration (*p* < 0.05), whereas moderate-intensity exercise elevated only SA1P (*p* < 0.001). Although knee extension increased muscle S1P content (*p* < 0.05), it was not released but taken up across the leg during exercise. However, sphingosine was released from both working and resting leg at the highest workload (*p* < 0.05).

**Conclusions:**

Plasma S1P concentration is elevated only by high-intensity exercise which results, at least in part, from increased availability of sphingosine released by skeletal muscle. In addition, exercise markedly affects S1P dynamics across the leg. We speculate that S1P may play an important role in adaptation of skeletal muscle to exercise.

**Electronic supplementary material:**

The online version of this article (doi:10.1007/s00421-014-3080-x) contains supplementary material, which is available to authorized users.

## Introduction

**Fig. 1:**
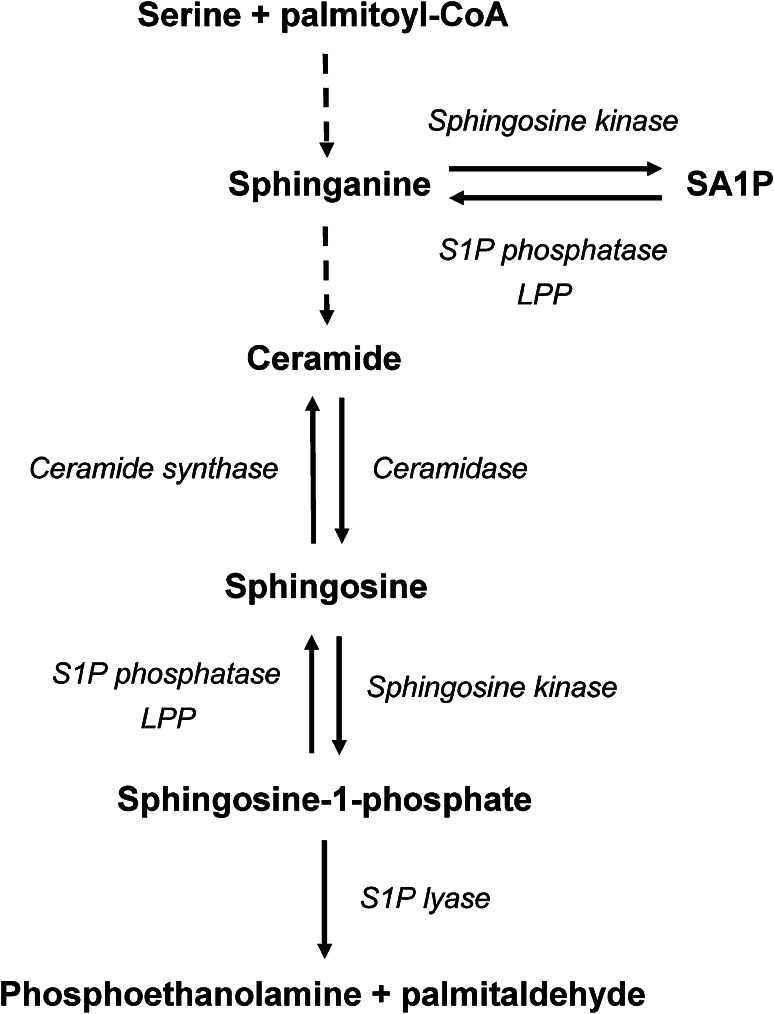
Schematic representation of metabolism of free sphingoid bases and their 1-phosphates. *LPP* lipid phosphate phosphatase, *S1P* sphingosine-1-phosphate, *SA1P* sphinganine-1-phosphate

Although sphingoid base-1-phosphates represent only a minor portion of the cellular sphingolipid pool, they are potent bioactive lipids. In mammals, sphingoid base-1-phosphates mainly include two compounds: sphingosine-1-phosphate (S1P) and sphinganine-1-phosphate (SA1P). Both S1P and SA1P are thought to be produced exclusively by phosphorylation of free sphingoid bases (sphingosine and sphinganine, respectively) catalyzed by sphingosine kinase (SPHK) (Liu et al. 2012). Substrates for sphingoid base-1-phosphates production can be provided through de novo synthesis, ceramide/dihydroceramide deacylation, or from extracellular sources (Gault et al. 2010). Degradation of sphingoid base-1-phosphates occurs either through the irreversible action of sphingosine-1-phosphate lyase to produce palmitaldehyde and phosphoethanolamine, or through dephosphorylation to free sphingoid bases by sphingosine-1-phosphate phosphatases and nonspecific lipid phosphatases (Liu et al. 2012) (Fig. 1).

S1P plays a dual role in cellular signaling, acting either as an intracellular messenger or by binding to a family of five plasma membrane G protein-coupled receptors (S1PRs) that are ubiquitously expressed in tissues (Strub et al. 2010). It should be noted that S1PRs are also activated by SA1P; however, the biological activity of this compound is poorly characterized (Tamama et al. 2001). S1P and SA1P are normal constituents found in human plasma in relatively high concentrations that are above the EC_50_ for S1PRs (Yatomi 2008). Studies conducted during the last few years revealed important physiological function of blood S1P. Pappu et al. (2007) found that high plasma concentration of this sphingolipid is required for lymphocyte egress from lymphoid organs to blood. In addition, mice that selectively lack S1P in plasma are characterized by increased basal and inflammation-induced vascular leak indicating impaired endothelial barrier function (Camerer et al. 2009). Plasma S1P is also involved in the regulation of vascular tone and cardiac function (Alewijnse et al. 2004), as well as bone homeostasis (Ishii and Kikuta 2013).

We have previously reported that exercise increased sphingoid base-1-phosphate levels in rat skeletal and cardiac muscle (Baranowski et al. 2008; Blachnio-Zabielska et al. 2008), which prompted us to examine in the subsequent study whether physical effort induces similar effects also in blood. We found that 60-min cycling exercise at 70 % of *V*O_2 max_ increased plasma levels of S1P and SA1P in untrained but not in endurance-trained subjects (Baranowski et al. 2011). On the other hand, we also showed that 48-hour ultramarathon run markedly reduced concentration of these sphingoid base-1-phosphates (Baranowski et al. 2014). These contradictory findings prompted us to further examine this issue in the present study by investigating the effect of exercise of various duration and intensity. Plasma S1P is implicated in several diseases, including cancer, myocardial infarction, osteoporosis and atherosclerosis (Strub et al. 2010; Hla and Brinkmann 2011). Therefore, clarification of the direction of exercise-induced change in plasma S1P concentration may have potential clinical relevance.

There is some evidence suggesting that skeletal muscle may contribute to alterations in plasma sphingoid base-1-phosphate levels associated with exercise. In our previous studies, exercise was found to induce accumulation of sphingosine and sphinganine in rat muscles in a time-dependent manner (Dobrzyn and Gorski 2002), and it should be noted that muscle tissue is able to release free sphingoid bases into the circulation (Cavalli et al. 2002). We hypothesized that blood during its passage through the working skeletal muscle is enriched with sphingosine and sphinganine, which are subsequently used by blood cells to produce S1P and SA1P, and that this effect depends on exercise intensity and/or duration. In the present study, we aimed to test this hypothesis by using two types of physical effort: rowing that engages all major muscle groups in the body, and one-leg knee extension exercise model that relies on work of isolated muscle group and lowers the strain on the cardiovascular system.

## Materials and methods

The investigation conforms with the principles outlined in the Declaration of Helsinki and was approved by the Ethical Committee for Human Studies of the Józef Piłsudski University of Physical Education and by the Ethics Committee for Medical Research in Copenhagen. All subjects gave their informed consent prior to their inclusion in the study.

### Exercise on the rowing ergometer

Thirteen healthy male athletes were recruited from the students of the Faculty of Physical Education and Sport at the Józef Piłsudski University of Physical Education. All subjects were non-smokers and were not taking any medicine at the time of enrollment. Ten of the subjects were runners, two were mixed martial arts competitors, and one was a cross-country skier (average experience of 5.8 ± 0.9 years). Characteristics of the subjects are given in Table 1. During the 4 weeks preceding the experiment the subjects performed eight 1-hour training sessions to familiarize with the rowing ergometer (Concept 2, model C, Morrisville, USA) and the proper technique of rowing. The participants were studied on two separate days over a 1-week period. On the first day, they were subjected to a graded exercise on the rowing ergometer starting at 100 W for 3 min followed by increments of 40 W every 3 min until exhaustion. Peak oxygen uptake (*V*O_2 max_) was determined during this session using an online system (Start 2000, MES, Cracow, Poland). Blood samples were drawn before and immediately after the exercise through a catheter inserted into an antecubital vein. The average time until exhaustion was 18.7 ± 0.6 min.

**Table 1:** Participant characteristics

	One-leg knee extension exercise (*n* = 10)	Exercise on the rowing ergometer (*n* = 13)
Age (years)	26 ± 2	22 ± 0.4
Height (cm)	183 ± 2	180 ± 2
BMI (kg/m^2^)	24.5 ± 0.8	22.6 ± 0.6
*V*O_2 max_ (ml/kg/min)	49 ± 2	56 ± 1

The results are mean ± SEM

Seven days later the subjects performed a 60-min exercise on the same rowing ergometer at a workload estimated to elicit a load of 65 % of individual *V*O_2 max_. The mean heart rate at 30 and 60 min of the exercise was 157 ± 4 and 157 ± 5, respectively. This time blood samples were taken just before the exercise, after 30 and 60 min of rowing, and following 30 min and 24 h of rest. Subjects were instructed to abstain from alcohol use during the 2 days preceding each visit in the laboratory.

### One-leg knee extension exercise

Ten healthy males were recruited for the study. Characteristics of the subjects are given in Table 1. This study and the basal data of the included subjects have been used for a prior publication with a different focus (Stallknecht et al. 2007). Prior to the experiment, subjects were accustomed to exercise on the knee extension ergometer and maximal work capacity (*W*_max_) was determined for each leg as described by Andersen and Saltin (1985). At least 5 days before the first experimental day, subjects performed a *V*O_2 max_ test on a cycle ergometer using a standard progressive exercise test. Subjects were instructed to abstain from alcohol and tobacco use on the days preceding each visit in the laboratory. On the day of the experiment, after an initial short rest, a catheter (Arterial Cannula with FloSwitch, Becton–Dickinson, UK) was inserted in the brachial or radial artery for blood sampling. Furthermore, catheters were placed in both femoral veins under local anesthesia by an aseptic technique, and the tips were advanced to ~2 cm below the inguinal ligament in the antegrade direction. The catheters were kept patent by intermittent flushing with sterile sodium citrate throughout the procedure. The protocol consisted of an initial 15-min resting period and three consecutive periods of one-leg knee extension exercise (the kicking rate was 60 times per minute). Subjects first exercised with one leg for 30 min at 25 % of *W*_max_ and after a 30-min rest subjects exercised with the other leg for 120 min at 55 % of *W*_max_ with a short break (~5 min, required to perform muscle biopsy) after the first 30 min. After a 30-min rest, the subjects again exercised with the first leg for 30 min but now at 85 % of *W*_max_. Selection of the leg eligible for low/high or moderate intensity was done by randomized stratification, such that dominant and non-dominant legs were similarly represented. Both during the resting and the exercise periods, subjects were sitting in a chair with the torso strapped to the back of the chair. Subjects had free access to water throughout the experiment. Prior to each exercise bout and during exercise, blood flow was measured by the Doppler technique as previously described (Radegran and Saltin 1998).

Before and after each exercise period (and after 30 min of exercise at 55 % of *W*_max_), a muscle biopsy was obtained from vastus lateralis in the working leg under local analgesia (2–3 ml lidocaine, 20 mg/ml) using the Bergström technique applied with suction. The incisions were made approximately 12–18 cm above the knee and two samples were obtained from each incision, but in the opposite direction, distal–proximal to that of the first biopsy. The biopsies were frozen in liquid nitrogen within 10–15 s of sampling and were stored at −80 °C until further analysis.

Blood was sampled at the same time points as the muscle biopsies were taken. Prior to each blood sample (15–20 s), a cuff placed under the knee was inflated to suprasystolic pressure to minimize contribution from the lower leg. Blood was sampled into iced tubes and immediately centrifuged to separate plasma.

Uptake and release of sphingolipids over the leg were calculated from arterial and femoral venous differences multiplied by plasma flow, according to the Fick principle.

### Blood fractionation

Immediately after sampling into 4 ml EDTA tubes, blood was centrifuged at 300×*g* for 10 min at 4 °C, and the platelet-rich plasma was transferred to a fresh plastic tube. The leukocyte-rich buffy coat was thoroughly removed. Separated erythrocytes were suspended in precooled phosphate-buffered saline (PBS), centrifuged at 800×*g* for 10 min at 4 °C and the upper layer as well as the remaining buffy coat was discarded. Red blood cells were then resuspended in PBS and flash frozen in liquid nitrogen. Platelet-rich plasma was centrifuged at 1,000×*g* for 10 min at 4 °C to separate platelets. Supernatant was then transferred to a fresh plastic tube and recentrifuged at 5,000×*g* for 10 min at 4 °C to obtain platelet-free plasma. Isolated thrombocytes were washed with platelet wash buffer (5 mM KH_2_PO_4_, 5 mM Na_2_HPO_4_, 0.1 M NaCl, 1 % glucose, 0.63 % sodium citrate, pH 6.6), suspended in PBS, and flash frozen in liquid nitrogen. All samples were stored at −80 °C until analysis.

Hemoglobin concentration in erythrocyte suspensions was determined colorimetrically using Drabkin’s reagent kit (Sigma, Schnelldorf, Germany). Protein concentration in platelet samples was measured with the BCA protein assay kit (Sigma). Bovine serum albumin (fatty acid free, Sigma) was used as a standard.

### Blood and muscle sphingolipid analysis

Standards utilizing 18C-sphingoid bases: sphingosine d18:1, sphinganine d18:0, sphingosine-1-phosphate d18:1, sphinganine-1-phosphate d18:0, d18:1/14:0-Cer—ceramide containing myristic acid (C14:0-Cer), d18:1/16:0-Cer—ceramide containing palmitic acid (C16:0-Cer), d18:1/17:0-Cer—ceramide containing margaric acid (C17:0-Cer, internal standard for ceramides), d18:1/18:0-Cer–ceramide containing stearic acid (C18:0-Cer), d18:1/18:1-Cer–ceramide containing oleic acid (C18:1-Cer), d18:1/20:0-Cer–ceramide containing arachidic acid (C20:0-Cer), d18:1/C22:0-Cer–ceramide containing behenic acid (C22:0-Cer), d18:1/24:0-Cer–ceramide containing lignoceric acid (C24:0-Cer), d18:1/24:1-Cer–ceramide containing nervonic acid (C24:1-Cer) as well as standards utilizing 17C-sphingoid bases: sphingosine (d17:1-Sph, internal standard for sphingosine and sphinganine), sphingosine-1-phosphate (d17:1-S1P, internal standard for S1P and SA1P), were purchased from Avanti Polar Lipids (Alabaster, AL). The HPLC-grade methanol and water as well as formic acid, ammonium formate and ethanol were obtained from Sigma–Aldrich (St. Louis, MO).

Sphingolipid content was determined using the method described by Blachnio-Zabielska et al. (2012) with minor modifications. In brief, sphingolipids were extracted from ~20 mg of tissue, 100 μl of plasma and erythrocyte suspension, and 150 μl of platelet suspension by the use of the extraction mixture composed of isopropanol:water:ethyl acetate (35:5:60; v:v:v). Quantitative measurement of sphingolipids (sphingosine, sphinganine, S1P, SA1P, C14:0-Cer, C16:0-Cer, C18:1-Cer, C18:0-Cer, C20:0-Cer, C22:0-Cer, C24:1-Cer, C24:0-Cer) was made using an Agilent 6460 Triple quadrupole mass spectrometer. Sphingolipids were analyzed using positive ion electrospray ionization source with selected reaction monitoring. The chromatographic separation was performed using an Agilent 1290 Infinity Ultra High Performance Liquid Chromatography system. The analytical column was a reverse-phase Zorbax SB-C8 column 2.1 × 150 mm, 1.8 μm (Agilent, Santa Clara, CA).

Chromatographic separation was conducted in binary gradient using 2 mM ammonium formate, 0.15 % formic acid in methanol as Solvent A and 1.5 mM ammonium formate, 0.1 % formic acid in water as Solvent B at the flow rate of 0.4 ml/min. C17-sphingosine, C17-S1P and C17:0-ceramide were used as an internal standards.

### Statistical analysis

All data are presented as mean ± SEM. Statistical comparisons were made using one-way repeated measures ANOVA followed by Newman–Keuls post hoc test or by Student’s *t* test for paired samples. *p* < 0.05 was considered statistically significant.

## Results

### Exercise on the rowing ergometer

**Fig. 2:**
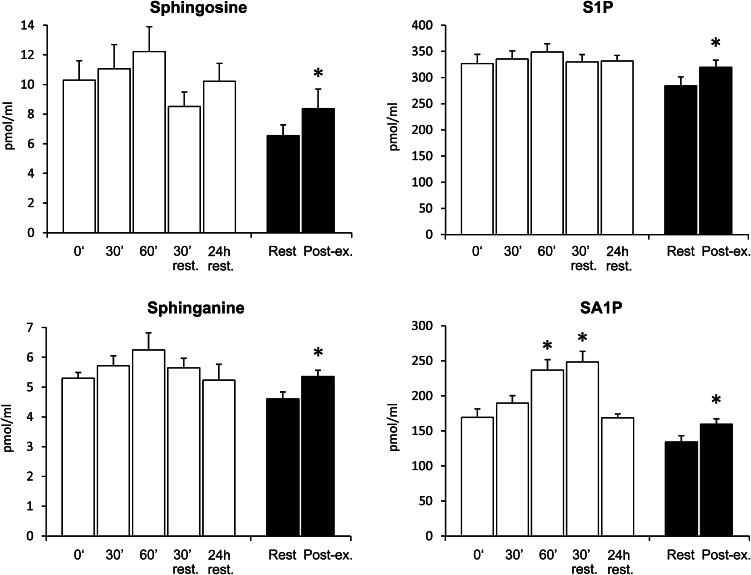
Effect of exercise on a rowing ergometer on sphingolipid concentration in the plasma. Subjects performed 60-min exercise at 65 % of individual *V*O_2 max_ (*white bars*) and graded exercise until exhaustion (*black bars*) on two separate days. Blood samples were taken from the antecubital vein at indicated time points (*n* = 13). The results are mean ± SEM. *p < 0.05 vs. the value before the respective exercise. *S1P* sphingosine-1-phosphate, *SA1P* sphinganine-1-phosphate

After 60 min of the exercise at 65 % of individual *V*O_2 max_ plasma, SA1P level was increased by 40 % (*p* < 0.01). This effect persisted for at least 30 min after cessation of the exercise (*p* < 0.001). However, 24 h later, plasma SA1P concentration was back to the baseline level. Exercise until exhaustion increased plasma sphingosine, sphinganine, S1P and SA1P concentration (by 27, 17, 12 and 19 %, respectively, *p* < 0.05) (Fig. 2). There were no significant changes in the total ceramide concentration or individual ceramide species levels during or after either exercise (see supplementary Table 1).

**Fig. 3:**
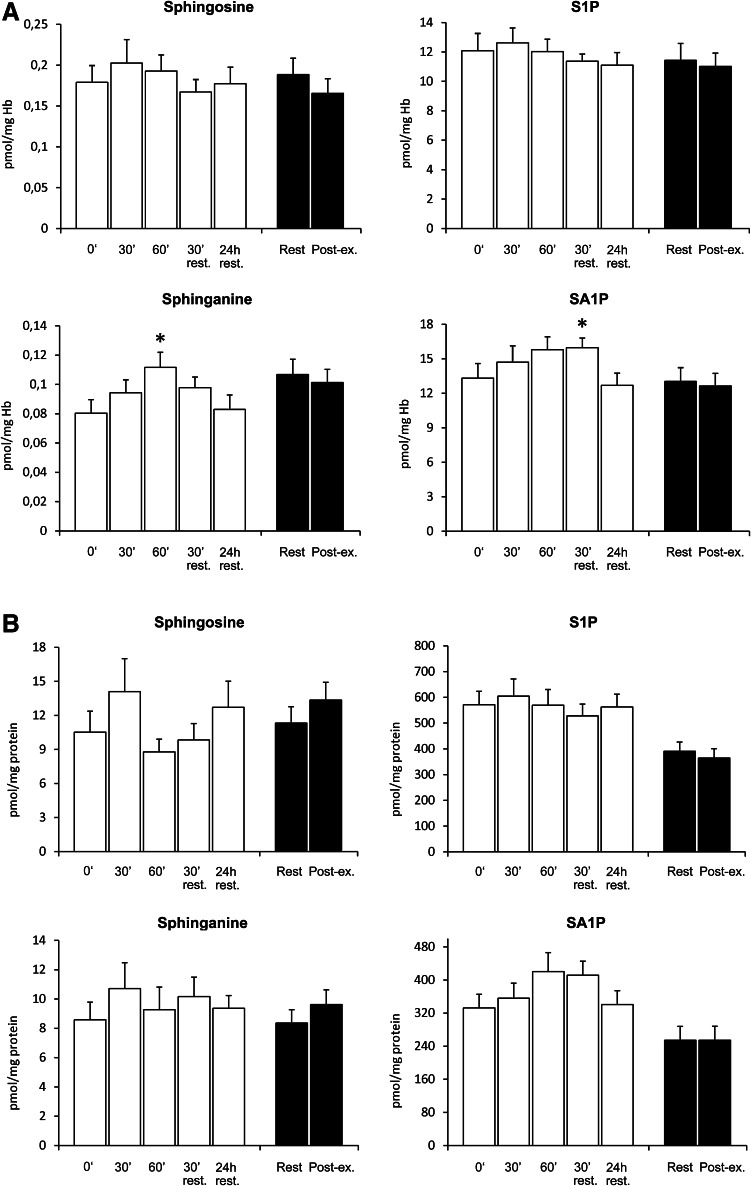
Effect of exercise on a rowing ergometer on erythrocyte (**a**) and platelet (**b**) sphingolipid content. Subjects performed 60-min exercise at 65 % of individual *V*O_2 max_ (*white bars*) and graded exercise until exhaustion (*black bars*) on two separate days. Blood samples were taken from the antecubital vein at indicated time points (*n* = 13). The results are mean ± SEM. *p < 0.05 vs. the value before the respective exercise. *S1P* sphingosine-1-phosphate, *SA1P* sphinganine-1-phosphate

The level of sphinganine in erythrocytes was increased by 39 % after 60 min of rowing at 65 % of individual *V*O_2 max_ (*p* < 0.01). A similar trend was observed for SA1P; however, the difference reached statistical significance only 30 min post-exercise (*p* < 0.05). Nevertheless, 24 h after cessation of the exercise, the content of both sphinganine and SA1P in erythrocytes decreased back to the initial value. Exercise until exhaustion did not affect erythrocyte level of free sphingoid bases or their 1-phosphates (Fig. 3a). Platelet sphingolipid levels were not significantly altered by either exercise (Fig. 3b). Neither individual ceramide species nor total ceramide level in erythrocytes and platelets was affected by exercise (see supplementary Tables 2 and 3).

### One-leg knee extension exercise

**Fig. 4:**
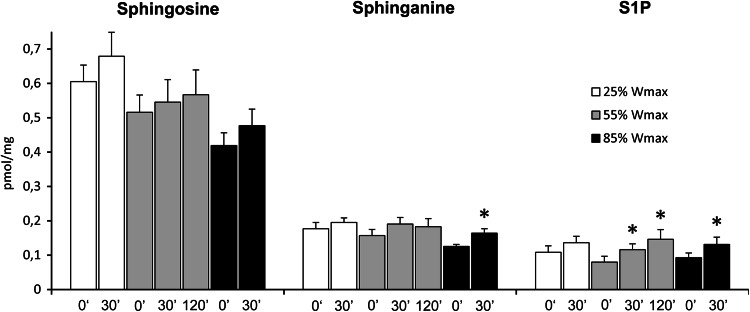
Effect of one-leg knee extension exercise on sphingolipid content in skeletal muscle. Subjects performed three consecutive periods of exercise separated by 30 min of rest. First subjects exercised with one leg for 30 min at 25 % of maximal workload (*W*_max_), then with the other leg for 120 min at 55 % of *W*_max_, and finally again with the first leg for 30 min at 85 % of *W*_max_. The biopsies of vastus lateralis muscle from the working leg were taken at indicated time points (*n* = 10). The results are mean ± SEM. *p < 0.05 vs. the value before the respective exercise. *S1P* sphingosine-1-phosphate

Muscle sphingosine and sphinganine content tended to increase after each exercise session, but the increase reached statistical significance only for sphinganine after exercise at the highest workload (31 % increase, *p* < 0.02). On the other hand, muscle S1P level was markedly elevated by exercise at 55 and 85 % of *W*_max_ (*p* < 0.05). In the former case, the content of S1P was increased both after 30 and 120 min of the exercise (by 45 and 82 %, respectively) (Fig. 4). The level of total ceramide or individual ceramide species in skeletal muscle was not significantly affected by exercise at either workload (see supplementary Table 4).

**Fig. 5:**
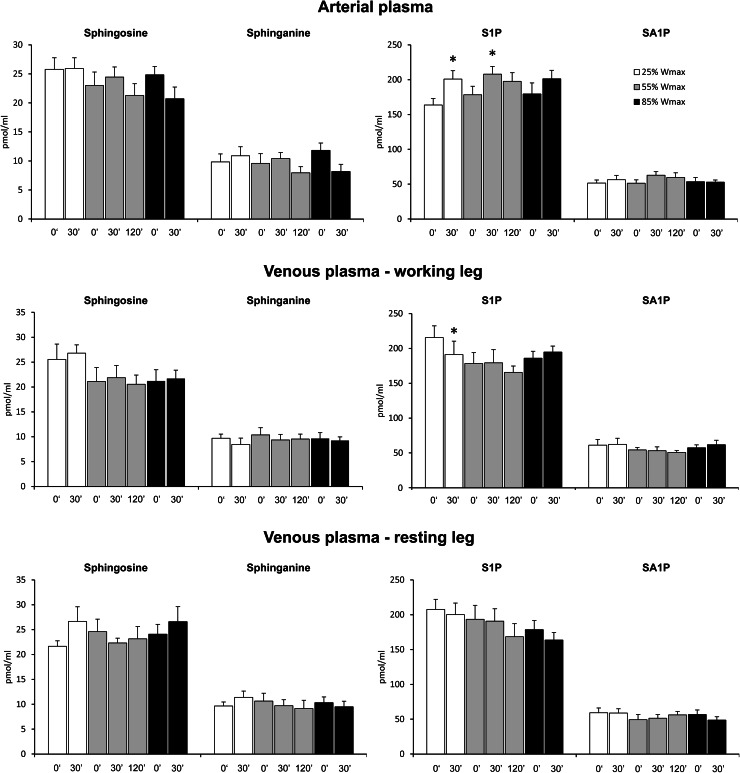
Effect of one-leg knee extension exercise on sphingolipid concentration in the plasma. Subjects performed three consecutive periods of exercise separated by 30 min of rest. First subjects exercised with one leg for 30 min at 25 % of maximal workload (*W*_max_), then with the other leg for 120 min at 55 % of *W*_max_, and finally again with the first leg for 30 min at 85 % of *W*_max_. Blood samples were taken from the radial artery as well as from both femoral veins at indicated time points (*n* = 10). The results are mean ± SEM. *p < 0.05 vs. the value before the respective exercise. *S1P* sphingosine-1-phosphate, *SA1P* sphinganine-1-phosphate

Basal S1P concentration in the venous plasma was 30 % higher than in the artery (213 ± 11 and 164 ± 9 pmol/ml, respectively, *p* < 0.01). Exercise tended to increase arterial S1P concentration. However, the difference reached statistical significance only in the case of exercise at 25 % of *W*_max_ and after 30 min of exercise at 55 % of *W*_max_ (*p* < 0.03). On the other hand, S1P concentration in the venous plasma taken from the working leg (but not from the resting one) was decreased after exercise at 25 % of *W*_max_ (*p* < 0.02, Fig. 5). The concentrations of total ceramide or individual ceramide species in the arterial as well as in the venous plasma were not affected by either exercise (see supplementary Tables 5–7).

**Fig. 6:**
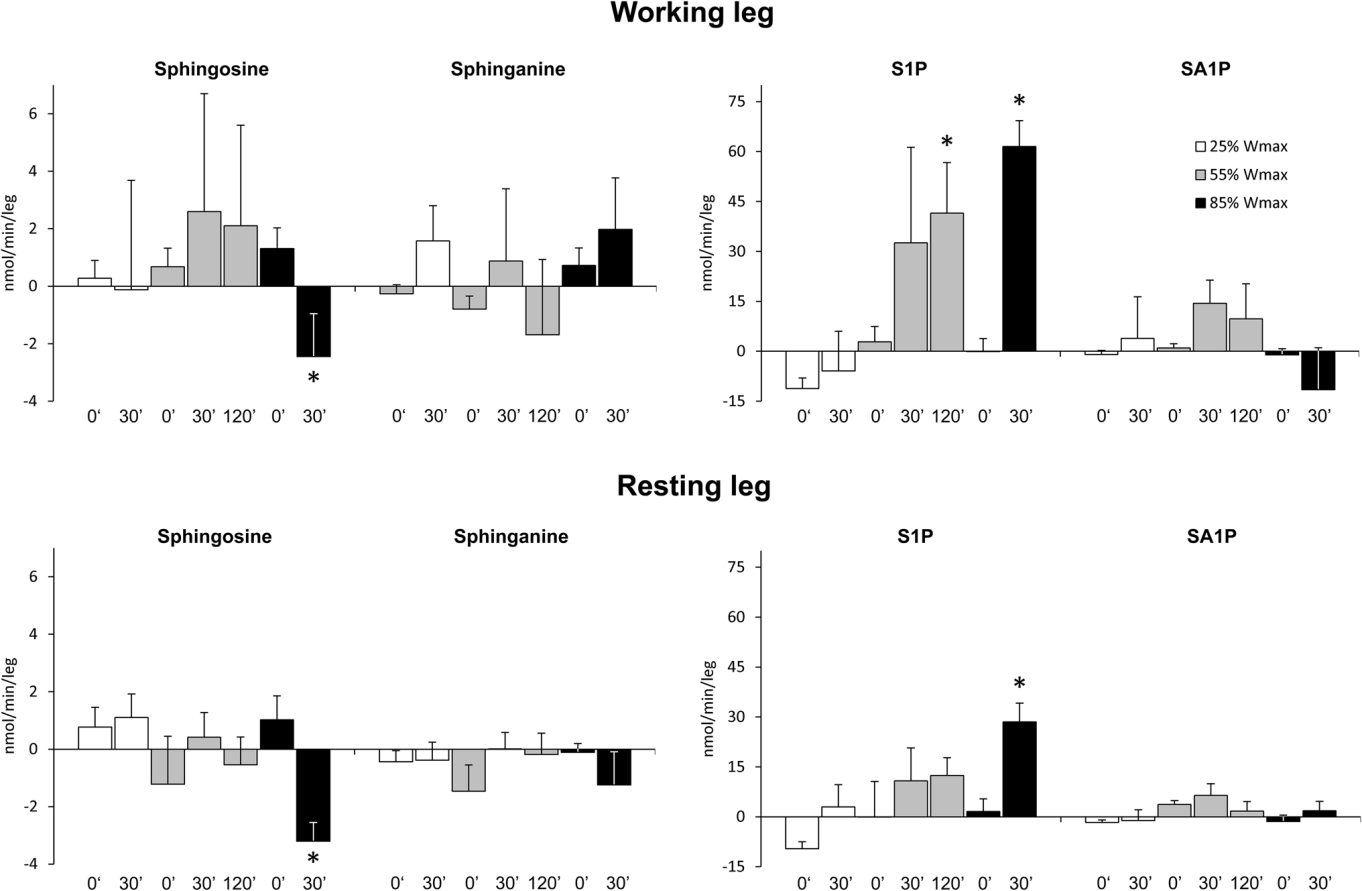
Effect of one-leg knee extension exercise on the rate of sphingolipid uptake (positive values) or release (negative values) across the leg. Subjects performed three consecutive periods of exercise separated by 30 min of rest. First subjects exercised with one leg for 30 min at 25 % of maximal workload (*W*_max_), then with the other leg for 120 min at 55 % of *W*_max_, and finally again with the first leg for 30 min at 85 % of *W*_max_. Blood samples were taken from the radial artery as well as from both femoral veins at indicated time points (*n* = 10). Uptake and release of sphingolipids over the leg were calculated from arterial and femoral venous differences multiplied by plasma flow, according to the Fick principle. The results are mean ± SEM. *p < 0.05 vs. the value before the respective exercise. *S1P* sphingosine-1-phosphate, *SA1P* sphinganine-1-phosphate

The rate of uptake/release of sphingolipids over the leg was calculated by multiplying the arterio-venous differences by plasma flow. Under basal conditions, S1P was released across both legs (Fig. 6). Surprisingly, after exercise at 55 and 85 % of *W*_max_, a marked uptake of S1P across the working leg was observed (*p* < 0.04 and *p* < 0.001, respectively). A similar, albeit much weaker trend could be seen also in the resting leg (*p* < 0.002, Fig. 6). It should be noted that after 30 min of rest separating each exercise, S1P was neither released nor taken up across either leg. Exercise at 85 % of *W*_max_ (but not at lower workloads) induced sphingosine release across both resting and working leg (*p* < 0.002 and *p* < 0.03, respectively). In the case of sphinganine and SA1P (Fig. 6), as well as total ceramide or individual ceramide species (see supplementary Tables 8 and 9), neither release nor uptake could be clearly observed under basal conditions or during exercise.

## Discussion

There is some evidence indicating that free sphingoid bases may affect muscle excitation–contraction coupling. Sabbadini et al. (1992) showed that sphingosine acts directly on the sarcoplasmic reticulum ryanodine receptor to inhibit calcium release in isolated rabbit muscle fibers. A similar, albeit weaker, effect is exerted by sphinganine (McDonough et al. 1994). It was also postulated that exercise-induced sphingosine production contributes to the development of muscle fatigue (Sabbadini et al. 1999). In our previous reports, marked accumulation of sphingosine and sphinganine was observed in rat skeletal muscles in response to electrical stimulation or exercise on a treadmill (Dobrzyn and Gorski 2002; Blachnio-Zabielska et al. 2008). However, in the present study, knee extension exercise induced only minor changes in muscle content of these sphingoid bases. Our results indicate that sphingosine and sphinganine are unlikely to play a significant role in skeletal muscle fatigue or regulation of muscle performance in humans. This is also supported by the fact that muscle and plasma-free sphingoid base levels did not correlate with *V*O_2 max_ regardless of the exercise intensity or duration (data not shown).

On the other hand, muscle S1P content was increased by knee extension exercise in proportion to its duration and intensity. This observation is in line with the results of our previous animal experiment (Blachnio-Zabielska et al. 2008). It was also recently reported that resistance training increases plasma S1P concentration and muscle S1PRs expression in rats (Banitalebi et al. 2013). There is a growing body of evidence from animal studies that S1P plays a significant role in skeletal muscle biology. Extracellular S1P was found to activate muscle satellite cells, stimulate myoblast differentiation, promote muscle repair, and exert a trophic effect on skeletal muscle. In addition, it was shown to affect the excitation–contraction coupling and delay muscle fatigue (reviewed in Donati et al. 2013). Taking together, our finding that exercise increases S1P level in both muscle and plasma suggests that this sphingolipid may play an important role in acute and/or long-term adaptation of skeletal muscle to exercise in humans. This is also supported by the fact that longer and more intense exercise, that is more likely to induce muscle fatigue, transient microdamage or hypertrophic response, was associated with greater increase in S1P level. However, further studies using genetic or pharmacological tools to manipulate S1P level in plasma and skeletal muscle are required to elucidate the role of S1P in exercise physiology. Mutant mice engineered to selectively lack S1P in plasma represent a perfect model for this purpose (Camerer et al. 2009).

We have previously found that prolonged, moderate-intensity exercise did not affect plasma sphingolipid levels in endurance-trained athletes (Baranowski et al. 2011). This is in contrast to the present study where increased plasma SA1P concentration was observed in trained subjects during and after exercise at 65 % of individual *V*O_2 max_. Duration and intensity of the exercise as well as subjects’ characteristics in these two studies are very similar. The only significant difference is that in the present report exercise was performed on a rowing ergometer, whereas in the previous one cycle ergometer was used. Cycling involves predominantly hip extensors, knee extensors and ankle plantar flexors (Ericson 1986), whereas rowing engages most of the principal muscle groups of both upper and lower body (Secher 1993). In consequence, a much larger fraction of the total muscle mass is recruited during rowing when compared to cycling exercise (∼30 kg vs. ∼15 kg in a 70 kg male) (Secher 1993). The fact that exercise engaging more muscle groups induced stronger effect on plasma SA1P level suggests that skeletal muscle contributed to this effect.

Red blood cells are considered to be an important source of plasma sphingoid base-1-phosphates since, in contrast to thrombocytes, they were shown to release S1P and SA1P spontaneously without any stimulation (Hanel et al. 2007). Interestingly, the increase in plasma SA1P concentration induced by exercise at 65 % of individual *V*O_2 max_ was associated with elevated sphinganine and SA1P content in erythrocytes. It is, therefore, most likely that the above-mentioned increase in plasma SA1P concentration resulted from enhanced synthesis and release of this sphingoid base-1-phosphate by red blood cells. However, the source of sphinganine that accumulated in erythrocytes remains obscure. Although we have observed a moderate increase in muscle sphinganine level after knee extension exercise at 85 % of *W*_max_, we did not find any evidence that it was released to the circulation.

Interestingly, graded exercise until exhaustion increased plasma concentration of both S1P and SA1P. This observation indicates that S1P level in the plasma is elevated only by very high-intensity exercise. The above effect was associated with increased plasma sphingosine and sphinganine concentration. This implies that elevation of the level of sphingoid base-1-phosphates might result from an increased availability of substrates required for their synthesis. Nevertheless, sphingolipid levels in erythrocytes and platelets were not affected by graded exercise until exhaustion which suggests that blood cells did not contribute to the effect observed in the plasma. Recent studies identified vascular endothelium and liver as other important contributors of plasma S1P (Venkataraman et al. 2008; Kurano et al. 2013). We speculate that graded exercise until exhaustion might have increased the rate of sphingoid base-1-phosphates production by these sources. It should be noted, however, that in the knee extension exercise experiment S1P and SA1P were not released across the leg at any workload (including the 30-minute rest period separating each exercise) which indicates that skeletal muscle did not directly contribute to elevated levels of sphingoid base-1-phosphates in plasma observed after exercise on a rowing ergometer. Nevertheless, we found that knee extension exercise at the highest workload induced sphingosine release across the leg suggesting that during intense work skeletal muscle may provide the substrate for increased S1P synthesis in other parts of the circulation. Interestingly, this phenomenon was observed in both working and resting leg suggesting that sphingosine release was not a direct consequence of muscle contractile activity but rather an effect of systemic changes related to the exercise.

Interestingly, in our study, basal S1P level was considerably lower in the arterial compared to the femoral vein plasma. This observation strongly indicates that S1P is released to skeletal muscle circulation at rest, most likely by the vascular endothelium. Surprisingly, knee extension exercise induced a drastic change in S1P metabolism. After exercise at the medium and high workload S1P was no longer released but taken up across the working leg. Interestingly, SA1P metabolism in muscle was not similarly affected, which provides an explanation why plasma level of this sphingoid base-1-phosphate is more likely to increase in response to exercise. It was reported that vascular endothelium is capable of not only secreting, but also degrading extracellular S1P. Intravenously administered C17–S1P is quickly eliminated from the plasma in mice (Salous et al. 2013), and in vitro experiments showed that extracellular S1P is rapidly degraded by human vascular endothelial cells via the action of lipid phosphate phosphatase expressed on the plasma membrane (Aoki et al. 2005; Zhao et al. 2007). Our results suggest that exercise alters the balance between S1P release and degradation in muscle vascular endothelium.

The major limitation of our study is that the training sessions preceding the experiment could possibly induce a training effect in athletes not accustomed to rowing. However, we believe that this effect (if any) was modest, since these sessions were of low intensity and the subjects were already highly endurance trained. The training sessions were necessary as preliminary tests showed that subjects who are not accustomed to rowing need to be taught a proper technique to be able to complete 1-hour exercise on a rowing ergometer.

## Conclusions

In summary, we found that under basal conditions S1P was released across the leg as the concentration of the compound was lower in the arterial compared to the femoral vein plasma. Exercise until exhaustion increased plasma concentration of S1P and SA1P, whereas moderate-intensity exercise elevated only SA1P level. Although knee extension exercise markedly increased muscle S1P content, sphingoid base-1-phosphates were not released but taken up across the leg during the exercise. It should be noted, however, that at the highest workload, sphingosine was released from both working and resting leg. We conclude that muscle S1P content is increased in proportion to exercise duration and intensity, whereas plasma S1P level is elevated only by very high-intensity exercise. The latter effect results, at least in part, from increased availability of sphingosine released by skeletal muscle. In addition, exercise markedly affects S1P dynamics across the leg that is manifested by a transition from release to uptake. We speculate that S1P may play an important role in adaptation of human skeletal muscle to exercise.

## Electronic supplementary material

Below is the link to the electronic supplementary material.

Supplementary material 1 (DOCX 42 kb)

## Abbreviations

ANOVA: Analysis of variance
PBS: Phosphate-buffered saline
S1P: Sphingosine-1-phosphate
S1PR: Sphingosine-1-phosphate receptor
SA1P: Sphinganine-1-phosphate
SPHK: Sphingosine kinase
*V*O_2 max_: Peak oxygen uptake
*W*_max_: Maximal work capacity

## References

[CR1] Alewijnse AE, Peters SL, Michel MC (2004). Cardiovascular effects of sphingosine-1-phosphate and other sphingomyelin metabolites. Br J Pharmacol.

[CR2] Andersen P, Saltin B (1985). Maximal perfusion of skeletal muscle in man. J Physiol.

[CR3] Aoki S, Yatomi Y, Ohta M, Osada M, Kazama F, Satoh K, Nakahara K, Ozaki Y (2005). Sphingosine 1-phosphate-related metabolism in the blood vessel. J Biochem.

[CR4] Banitalebi E, Gharakhanlou R, Ghatrehsamani K, Parnow AH, Teimori H, Mohammad Amoli M (2013). The effect of resistance training on plasma S1P level and gene expression of S1P1, 2, 3 receptors in male Wistar rats. Minerva Endocrinol.

[CR5] Baranowski M, Zabielski P, Blachnio A, Gorski J (2008). Effect of exercise duration on ceramide metabolism in the rat heart. Acta Physiol (Oxf).

[CR6] Baranowski M, Charmas M, Dlugolecka B, Gorski J (2011). Exercise increases plasma levels of sphingoid base-1 phosphates in humans. Acta Physiol (Oxf).

[CR7] Baranowski M, Gorski J, Klapcinska B, Waskiewicz Z, Sadowska-Krepa E (2014). Ultramarathon run markedly reduces plasma sphingosine-1-phosphate concentration. Int J Sport Nutr Exerc Metab.

[CR8] Blachnio-Zabielska A, Baranowski M, Zabielski P, Gorski J (2008). Effect of exercise duration on the key pathways of ceramide metabolism in rat skeletal muscles. J Cell Biochem.

[CR9] Blachnio-Zabielska AU, Persson XM, Koutsari C, Zabielski P, Jensen MD (2012). A liquid chromatography/tandem mass spectrometry method for measuring the in vivo incorporation of plasma free fatty acids into intramyocellular ceramides in humans. Rapid Commun Mass Spectrom.

[CR10] Camerer E, Regard JB, Cornelissen I, Srinivasan Y, Duong DN, Palmer D, Pham TH, Wong JS, Pappu R, Coughlin SR (2009). Sphingosine-1-phosphate in the plasma compartment regulates basal and inflammation-induced vascular leak in mice. J Clin Invest.

[CR11] Cavalli AL, Ligutti JA, Gellings NM, Castro E, Page MT, Klepper RE, Palade PT, McNutt WT, Sabbadini RA (2002). The role of TNFalpha and sphingolipid signaling in cardiac hypoxia: evidence that cardiomyocytes release TNFalpha and sphingosine. Basic Appl Myol.

[CR12] Dobrzyn A, Gorski J (2002). Effect of acute exercise on the content of free sphinganine and sphingosine in different skeletal muscle types of the rat. Horm Metab Res.

[CR13] Donati C, Cencetti F, Bruni P (2013). Sphingosine 1-phosphate axis: a new leader actor in skeletal muscle biology. Front Physiol.

[CR14] Ericson M (1986). On the biomechanics of cycling. A study of joint and muscle load during exercise on the bicycle ergometer. Scand J Rehabil Med Suppl.

[CR15] Gault CR, Obeid LM, Hannun YA (2010). An overview of sphingolipid metabolism: from synthesis to breakdown. Adv Exp Med Biol.

[CR16] Hanel P, Andreani P, Graler MH (2007). Erythrocytes store and release sphingosine 1-phosphate in blood. FASEB J.

[CR17] Hla T, Brinkmann V (2011). Sphingosine 1-phosphate (S1P): physiology and the effects of S1P receptor modulation. Neurology.

[CR18] Ishii M, Kikuta J (2013). Sphingosine-1-phosphate signaling controlling osteoclasts and bone homeostasis. Biochim Biophys Acta.

[CR19] Kurano M, Tsukamoto K, Ohkawa R, Hara M, Iino J, Kageyama Y, Ikeda H, Yatomi Y (2013). Liver involvement in sphingosine 1-phosphate dynamism revealed by adenoviral hepatic overexpression of apolipoprotein M. Atherosclerosis.

[CR20] Liu X, Zhang QH, Yi GH (2012). Regulation of metabolism and transport of sphingosine-1-phosphate in mammalian cells. Mol Cell Biochem.

[CR21] McDonough PM, Yasui K, Betto R, Salviati G, Glembotski CC, Palade PT, Sabbadini RA (1994). Control of cardiac Ca2+ levels. Inhibitory actions of sphingosine on Ca2+ transients and L-type Ca2+ channel conductance. Circ Res.

[CR22] Pappu R, Schwab SR, Cornelissen I, Pereira JP, Regard JB, Xu Y, Camerer E, Zheng YW, Huang Y, Cyster JG, Coughlin SR (2007). Promotion of lymphocyte egress into blood and lymph by distinct sources of sphingosine-1-phosphate. Science.

[CR23] Radegran G, Saltin B (1998). Muscle blood flow at onset of dynamic exercise in humans. Am J Physiol.

[CR24] Sabbadini RA, Betto R, Teresi A, Fachechi-Cassano G, Salviati G (1992). The effects of sphingosine on sarcoplasmic reticulum membrane calcium release. J Biol Chem.

[CR25] Sabbadini RA, Danieli-Betto D, Betto R (1999). The role of sphingolipids in the control of skeletal muscle function: a review. Ital J Neurol Sci.

[CR26] Salous AK, Panchatcharam M, Sunkara M, Mueller P, Dong A, Wang Y, Graf GA, Smyth SS, Morris AJ (2013). Mechanism of rapid elimination of lysophosphatidic acid and related lipids from the circulation of mice. J Lipid Res.

[CR27] Secher NH (1993). Physiological and biomechanical aspects of rowing. Implications for training. Sports Med.

[CR28] Stallknecht B, Dela F, Helge JW (2007). Are blood flow and lipolysis in subcutaneous adipose tissue influenced by contractions in adjacent muscles in humans?. Am J Physiol Endocrinol Metab.

[CR29] Strub GM, Maceyka M, Hait NC, Milstien S, Spiegel S (2010). Extracellular and intracellular actions of sphingosine-1-phosphate. Adv Exp Med Biol.

[CR30] Tamama K, Kon J, Sato K, Tomura H, Kuwabara A, Kimura T, Kanda T, Ohta H, Ui M, Kobayashi I, Okajima F (2001). Extracellular mechanism through the Edg family of receptors might be responsible for sphingosine-1-phosphate-induced regulation of DNA synthesis and migration of rat aortic smooth-muscle cells. Biochem J.

[CR31] Venkataraman K, Lee YM, Michaud J, Thangada S, Ai Y, Bonkovsky HL, Parikh NS, Habrukowich C, Hla T (2008). Vascular endothelium as a contributor of plasma sphingosine 1-phosphate. Circ Res.

[CR32] Yatomi Y (2008). Plasma sphingosine 1-phosphate metabolism and analysis. Biochim Biophys Acta.

[CR33] Zhao Y, Kalari SK, Usatyuk PV, Gorshkova I, He D, Watkins T, Brindley DN, Sun C, Bittman R, Garcia JG, Berdyshev EV, Natarajan V (2007). Intracellular generation of sphingosine 1-phosphate in human lung endothelial cells: role of lipid phosphate phosphatase-1 and sphingosine kinase 1. J Biol Chem.

