# Supplementary material for: Exercise increases sphingoid base-1-phosphate levels in human blood and skeletal muscle in a time- and intensity-dependent manner
Source: Eur J Appl Physiol. 2014 Dec 18;115(5):993–1003. doi: 10.1007/s00421-014-3080-x (PMC4412691; doi:10.1007/s00421-014-3080-x)
Supplement: Supplementary file 1 — Supplementary material 1 (DOCX 42 kb) [file 421_2014_3080_MOESM1_ESM.docx]

**Table 1**. Effect of exercise on a rowing ergometer on the concentration of ceramides in the plasma.

|  | Exercise at 65% of VO_2max_ | | | | |  | Exercise until exhaustion | |
| --- | --- | --- | --- | --- | --- | --- | --- | --- |
|  | 0’ | 30’ | 60’ | 30’ rest. | 24h rest. |  | Rest | Post-ex. |
| C14:0-Cer | 44,4±5,0 | 41,1±5,4 | 45,5±7,9 | 43,4±6,2 | 43,0±6,3 |  | 42,5±4,4 | 45,0±5,9 |
| C16:0-Cer | 766±113 | 739±75 | 768±86 | 753±73 | 723±89 |  | 598±40 | 658±75 |
| C18:1-Cer | 31,0±3,3 | 32,0±3,3 | 35,2±3,9 | 35,4±3,8 | 30,9±2,9 |  | 26,8±2,0 | 29,3±2,4 |
| C18:0-Cer | 21,9±3,2 | 18,9±2,0 | 22,0±3,0 | 21,7±3,7 | 29,3±5,9 |  | 17,3±1,2 | 18,6±1,6 |
| C20:0-Cer | 40,9±4,5 | 37,1±4,1 | 37,0±4,6 | 37,3±4,2 | 42,1±4,9 |  | 36,4±3,8 | 38,2±4,5 |
| C22:0-Cer | 779±124 | 750±104 | 744±117 | 707±107 | 721±84 |  | 628±74 | 693±110 |
| C24:1-Cer | 1058±162 | 1029±115 | 1028±145 | 939±107 | 1037±132 |  | 890±49 | 961±72 |
| C24:0-Cer | 4484±734 | 4103±597 | 4347±682 | 3940±438 | 4021±413 |  | 4172±470 | 4734±688 |
| Total | 7226±1150 | 6750±905 | 7028±1049 | 6477±743 | 6649±738 |  | 6412±644 | 7177±959 |

The results (pmol/ml) are means ± SEM. Subjects performed 60-minute exercise at 65% of individual VO_2 max_ and graded exercise until exhaustion on two separate days. Blood samples were taken from the antecubital vein at indicated time points (n=13).

**Table 2**. Effect of exercise on a rowing ergometer on the content of ceramides in erythrocytes.

|  | Exerciseat 65% of VO_2max_ | | | | |  | Exercise until exhaustion | |
| --- | --- | --- | --- | --- | --- | --- | --- | --- |
|  | 0’ | 30’ | 60’ | 30’ rest. | 24h rest. |  | Rest | Post-ex. |
| C14:0-Cer | 0,094±0,010 | 0,097±0,010 | 0,096±0,008 | 0,093±0,007 | 0,087±0,005 |  | 0,104±0,011 | 0,092±0,006 |
| C16:0-Cer | 6,96±0,76 | 6,40±0,75 | 6,65±0,63 | 6,00±0,44 | 6,24±0,65 |  | 6,56±0,60 | 5,74±0,53 |
| C18:1-Cer | 0,25±0,02 | 0,25±0,02 | 0,26±0,03 | 0,24±0,01 | 0,24±0,02 |  | 0,27±0,03 | 0,24±0,02 |
| C18:0-Cer | 1,07±0,13 | 1,01±0,18 | 1,07±0,13 | 0,95±0,10 | 1,08±0,14 |  | 1,12±0,11 | 0,94±0,11 |
| C20:0-Cer | 0,43±0,05 | 0,40±0,06 | 0,42±0,05 | 0,37±0,03 | 0,40±0,04 |  | 0,44±0,03 | 0,39±0,04 |
| C22:0-Cer | 2,46±0,31 | 2,32±0,29 | 2,44±0,29 | 2,13±0,18 | 2,26±0,24 |  | 2,79±0,21 | 2,36±0,25 |
| C24:1-Cer | 14,0±1,5 | 12,9±1,1 | 13,5±1,1 | 12,7±0,8 | 12,5±0,8 |  | 14,1±1,0 | 12,8±1,2 |
| C24:0-Cer | 7,24±0,84 | 6,83±0,77 | 7,25±0,80 | 6,57±0,54 | 6,81±0,73 |  | 8,48±0,59 | 6,90±0,77 |
| Total | 32,5±3,7 | 30,3±3,2 | 31,7±3,0 | 29,0±2,1 | 29,7±2,6 |  | 33,9±2,6 | 29,5±2,9 |

The results (pmol/mg Hb) are means ± SEM. Subjects performed 60-minute exercise at 65% of individual VO_2 max_ and graded exercise until exhaustion on two separate days. Blood samples were taken from the antecubital vein at indicated time points (n=13).

**Table 3**. Effect of exercise on a rowing ergometer on the content of ceramides in platelets.

|  | Exerciseat 65% of VO_2max_ | | | | |  | Exercise until exhaustion | |
| --- | --- | --- | --- | --- | --- | --- | --- | --- |
|  | 0’ | 30’ | 60’ | 30’ rest. | 24h rest. |  | Rest | Post-ex. |
| C14:0-Cer | 2,02±0,14 | 2,14±0,28 | 2,28±0,27 | 2,18±0,29 | 2,34±0,33 |  | 1,44±0,10 | 1,45±0,10 |
| C16:0-Cer | 100,9±8,5 | 96,7±11,4 | 96,6±7,1 | 101,8±8,7 | 102,9±3,6 |  | 70,8±5,8 | 73,7±5,8 |
| C18:1-Cer | 2,57±0,21 | 2,69±0,28 | 2,80±0,34 | 2,67±0,30 | 4,11±1,43 |  | 1,65±0,16 | 1,72±0,16 |
| C18:0-Cer | 33,7±8,6 | 45,6±14,0 | 26,3±5,8 | 23,3±4,9 | 46,6±7,7 |  | 16,5±3,4 | 16,2±3,8 |
| C20:0-Cer | 22,3±1,8 | 23,0±3,1 | 20,9±2,2 | 22,6±2,0 | 26,3±2,1 |  | 14,9±1,5 | 16,7±2,1 |
| C22:0-Cer | 71,4±8,3 | 68,3±7,9 | 67,0±7,9 | 69,8±7,4 | 78,8±5,7 |  | 51,9±3,7 | 56,8±4,6 |
| C24:1-Cer | 40,4±4,6 | 36,0±3,2 | 36,5±3,2 | 37,5±3,1 | 44,9±4,3 |  | 31,1±2,3 | 35,7±2,5 |
| C24:0-Cer | 103,5±10,4 | 94,4±10,5 | 101,2±11,0 | 101,7±8,8 | 108,4±7,6 |  | 70,2±4,3 | 74,2±3,5 |
| Total | 377±43 | 369±51 | 354±38 | 362±35 | 414±33 |  | 258±21 | 276±23 |

The results (pmol/mg protein) are means ± SEM. Subjects performed 60-minute exercise at 65% of individual VO_2 max_ and graded exercise until exhaustion on two separate days. Blood samples were taken from the antecubital vein at indicated time points (n=13).

**Table 4**. Effect of one leg knee extension exercise on the content of ceramides in skeletal muscle.

|  | 25% of Wmax | |  | 55% of Wmax | | |  | 85% of Wmax | |
| --- | --- | --- | --- | --- | --- | --- | --- | --- | --- |
|  | 0’ | 30’ |  | 0’ | 30’ | 120’ |  | 0’ | 30’ |
| C14:0-Cer | 0,076±0,009 | 0,071±0,004 |  | 0,062±0,006 | 0,064±0,006 | 0,064±0,007 |  | 0,060±0,003 | 0,059±0,005 |
| C16:0-Cer | 3,13±0,39 | 2,87±0,24 |  | 2,07±0,24 | 2,35±0,20 | 2,43±0,35 |  | 2,12±0,16 | 2,21±0,19 |
| C18:1-Cer | 0,82±0,05 | 0,86±0,07 |  | 0,91±0,08 | 0,93±0,13 | 0,86±0,09 |  | 0,86±0,06 | 0,78±0,07 |
| C18:0-Cer | 6,37±0,40 | 6,99±0,40 |  | 7,13±0,42 | 7,16±0,76 | 6,62±0,59 |  | 6,66±0,41 | 6,31±0,37 |
| C20:0-Cer | 0,13±0,02 | 0,13±0,01 |  | 0,10±0,01 | 0,11±0,01 | 0,11±0,01 |  | 0,10±0,01 | 0,11±0,01 |
| C22:0-Cer | 0,76±0,05 | 0,79±0,05 |  | 0,67±0,05 | 0,68±0,06 | 0,69±0,08 |  | 0,65±0,04 | 0,66±0,04 |
| C24:1-Cer | 1,42±0,06 | 1,60±0,11 |  | 1,38±0,09 | 1,45±0,12 | 1,32±0,11 |  | 1,28±0,09 | 1,29±0,07 |
| C24:0-Cer | 5,47±0,38 | 5,99±0,66 |  | 4,25±0,33 | 4,85±0,57 | 5,04±0,98 |  | 5,06±0,51 | 5,06±0,40 |
| Total | 18,2±1,0 | 19,3±1,0 |  | 16,6±1,0 | 17,6±1,6 | 17,1±1,9 |  | 16,8±1,1 | 16,5±0,9 |

The results (pmol/mg) are means ± SEM. Subjects performed three consecutive periods of exercise separated by 30min of rest. First subjects exercised with one leg for 30min at 25% of maximal workload (Wmax), then with the other leg for 120min at 55% of Wmax, and finally again with the first leg for 30min at 85% of Wmax. The biopsies of vastus lateralis muscle from the working leg were taken at indicated time points (n=10).

**Table 5**. Effect of one leg knee extension exercise on the concentration of ceramides in arterial plasma.

|  | 25% of Wmax | |  | 55% of Wmax | | |  | 85% of Wmax | |
| --- | --- | --- | --- | --- | --- | --- | --- | --- | --- |
|  | 0’ | 30’ |  | 0’ | 30’ | 120’ |  | 0’ | 30’ |
| C14:0-Cer | 6,21±0,39 | 7,03±0,91 |  | 6,60±1,04 | 8,57±1,21 | 7,39±0,76 |  | 7,58±1,25 | 7,96±1,36 |
| C16:0-Cer | 228±26 | 231±15 |  | 220±28 | 258±26 | 266±38 |  | 237±28 | 263±26 |
| C18:1-Cer | 3,83±0,44 | 3,99±0,44 |  | 3,91±0,43 | 4,17±0,40 | 4,28±0,57 |  | 4,16±0,43 | 4,79±0,45 |
| C18:0-Cer | 56,1±6,4 | 56,9±7,2 |  | 49,9±4,6 | 54,7±4,4 | 61,3±5,8 |  | 54,0±6,5 | 65,0±7,3 |
| C20:0-Cer | 63,7±12,2 | 52,4±8,5 |  | 47,8±5,4 | 47,5±6,4 | 54,6±8,6 |  | 47,3±6,2 | 54,9±12,7 |
| C22:0-Cer | 257±29 | 294±28 |  | 281±34 | 278±25 | 304±32 |  | 275±34 | 282±42 |
| C24:1-Cer | 540±36 | 560±23 |  | 541±37 | 511±27 | 550±24 |  | 490±25 | 522±41 |
| C24:0-Cer | 2720±58 | 2811±108 |  | 2770±66 | 2789±59 | 2885±66 |  | 2738±47 | 2647±137 |
| Total | 3898±111 | 4015±146 |  | 3921±98 | 3951±71 | 4134±67 |  | 3852±77 | 3859±205 |

The results (pmol/ml) are means ± SEM. Subjects performed three consecutive periods of exercise separated by 30min of rest. First subjects exercised with one leg for 30min at 25% of maximal workload (Wmax), then with the other leg for 120min at 55% of Wmax, and finally again with the first leg for 30min at 85% of Wmax. Blood samples were taken from the radial artery at indicated time points (n=10).

**Table 6**. Effect of one leg knee extension exercise on the concentration of ceramides in femoral vein plasma from the working leg.

|  | 25% of Wmax | |  | 55% of Wmax | | |  | 85% of Wmax | |
| --- | --- | --- | --- | --- | --- | --- | --- | --- | --- |
|  | 0’ | 30’ |  | 0’ | 30’ | 120’ |  | 0’ | 30’ |
| C14:0-Cer | 6,05±0,68 | 6,14±0,81 |  | 6,46±0,76 | 7,68±1,07 | 8,74±1,42 |  | 11,18±2,34 | 7,26±0,99 |
| C16:0-Cer | 191±28 | 208±44 |  | 180±23 | 231±21 | 230±21 |  | 233±44 | 228±30 |
| C18:1-Cer | 3,64±0,32 | 3,76±0,32 |  | 3,37±0,32 | 4,31±0,46 | 4,68±0,61 |  | 4,74±0,73 | 4,18±0,39 |
| C18:0-Cer | 59,3±9,4 | 56,8±7,4 |  | 53,2±7,1 | 61,5±5,4 | 60,3±4,3 |  | 70,5±7,9 | 76,8±8,7 |
| C20:0-Cer | 67,0±12,8 | 52,8±7,2 |  | 57,3±6,4 | 61,3±9,0 | 57,1±8,8 |  | 68,1±14,8 | 67,5±9,0 |
| C22:0-Cer | 306±35 | 326±28 |  | 293±40 | 294±19 | 323±32 |  | 346±37 | 368±35 |
| C24:1-Cer | 531±28 | 582±41 |  | 499±32 | 539±23 | 600±24 |  | 570±43 | 529±37 |
| C24:0-Cer | 2732±67 | 2649±73 |  | 2524±97 | 2740±81 | 2716±72 |  | 2719±96 | 2626±87 |
| Total | 3897±113 | 3886±115 |  | 3616±130 | 3939±79 | 4000±65 |  | 4022±165 | 3906±126 |

The results (pmol/ml) are means ± SEM. Subjects performed three consecutive periods of exercise separated by 30min of rest. First subjects exercised with one leg for 30min at 25% of maximal workload (Wmax), then with the other leg for 120min at 55% of Wmax, and finally again with the first leg for 30min at 85% of Wmax. Blood samples were taken from the femoral vein of the working leg at indicated time points (n=10).

**Table 7**. Effect of one leg knee extension exercise on the concentration of ceramides in femoral vein plasma from the resting leg.

|  | 25% of Wmax | |  | 55% of Wmax | | |  | 85% of Wmax | |
| --- | --- | --- | --- | --- | --- | --- | --- | --- | --- |
|  | 0’ | 30’ |  | 0’ | 30’ | 120’ |  | 0’ | 30’ |
| C14:0-Cer | 7,09±0,88 | 8,59±1,27 |  | 5,92±0,52 | 6,60±0,38 | 8,00±1,10 |  | 9,16±1,28 | 9,58±2,06 |
| C16:0-Cer | 211±22 | 240±25 |  | 201±26 | 293±55 | 244±25 |  | 262±37 | 251±42 |
| C18:1-Cer | 3,48±0,22 | 4,19±0,55 |  | 3,69±0,36 | 4,89±0,90 | 4,19±0,47 |  | 5,52±0,88 | 5,74±1,02 |
| C18:0-Cer | 54,8±5,1 | 55,8±6,2 |  | 55,7±8,3 | 58,2±4,8 | 55,3±5,6 |  | 64,5±5,6 | 72,0±8,6 |
| C20:0-Cer | 57,0±7,2 | 53,6±10,9 |  | 54,9±8,5 | 49,9±8,9 | 53,2±9,5 |  | 48,4±9,8 | 57,0±11,7 |
| C22:0-Cer | 325±33 | 263±32 |  | 316±32 | 331±37 | 300±31 |  | 311±34 | 306±30 |
| C24:1-Cer | 558±28 | 509±36 |  | 522±18 | 550±34 | 532±33 |  | 530±29 | 542±29 |
| C24:0-Cer | 2644±80 | 2678±96 |  | 2671±85 | 2716±143 | 2504±50 |  | 2789±81 | 2725±66 |
| Total | 3860±107 | 3813±145 |  | 3831±107 | 4013±200 | 3701±99 |  | 4022±147 | 3971±113 |

The results (pmol/ml) are means ± SEM. Subjects performed three consecutive periods of exercise separated by 30min of rest. First subjects exercised with one leg for 30min at 25% of maximal workload (Wmax), then with the other leg for 120min at 55% of Wmax, and finally again with the first leg for 30min at 85% of Wmax. Blood samples were taken from the femoral vein of the resting leg at indicated time points (n=10).

**Table 8**. Effect of one leg knee extension exercise on the rate of ceramide uptake (positive values) or release (negative values) across the working leg.

|  | 25% of Wmax | |  | 55% of Wmax | | |  | 85% of Wmax | |
| --- | --- | --- | --- | --- | --- | --- | --- | --- | --- |
|  | 0’ | 30’ |  | 0’ | 30’ | 120’ |  | 0’ | 30’ |
| C14:0-Cer | 0,08±0,15 | -0,07±1,67 |  | -0,05±0,13 | 1,53±1,37 | -2,19±1,94 |  | -0,93±0,52 | -0,26±1,54 |
| C16:0-Cer | 6,2±3,7 | 15,9±37,3 |  | 6,7±8,1 | 35,1±24,5 | 55,0±41,3 |  | 3,4±9,5 | 45,1±41,6 |
| C18:1-Cer | 0,04±0,05 | -0,01±0,54 |  | 0,12±0,05 | -0,22±0,36 | -0,49±0,48 |  | -0,13±0,13 | 0,32±0,82 |
| C18:0-Cer | 1,1±1,2 | 1,4±7,0 |  | 1,4±1,0 | -10,2±4,8 | -2,8±7,6 |  | -2,6±1,2 | -16,5±8,2 |
| C20:0-Cer | -1,9±2,0 | 1,0±10,2 |  | -0,1±0,8 | -22,1±9,6 | -15,2±15,2 |  | -2,5±2,4 | -12,3±18,4 |
| C22:0-Cer | -8,5±5,0 | -18,8±41,3 |  | -2,1±10,2 | -20,2±63,7 | -29,9±47,8 |  | -19,7±7,3 | -173,7±65,6 |
| C24:1-Cer | -2,4±14,3 | -23,8±28,2 |  | 24,0±16,5 | -48,3±59,5 | -62,4±70,3 |  | -23,2±8,6 | 34,4±79,6 |
| C24:0-Cer | 17,8±26,7 | 316,8±120,1 |  | 83,9±47,3 | 70,6±184,7 | 207,7±167,0 |  | 17,0±29,5 | 300,4±294,9 |
| Total | 12,4±33,3 | 292,4±168,7 |  | 113,9±58,5 | 6,4±185,2 | 154,2±135,6 |  | -28,6±38,3 | 207,2±355,7 |

The results (nmol/min/leg) are means ± SEM. Subjects performed three consecutive periods of exercise separated by 30min of rest. First subjects exercised with one leg for 30min at 25% of maximal workload (Wmax), then with the other leg for 120min at 55% of Wmax, and finally again with the first leg for 30min at 85% of Wmax. Blood samples were taken from the radial artery as well as from the femoral vein of the working leg at indicated time points (n=10). Uptake and release of sphingolipids over the leg were calculated from arterial and femoral venous differences multiplied by plasma flow, according to the Fick principle.

**Table 9**. Effect of one leg knee extension exercise on the rate of ceramide uptake (positive values) or release (negative values) across the resting leg.

|  | 25% of Wmax | |  | 55% of Wmax | | |  | 85% of Wmax | |
| --- | --- | --- | --- | --- | --- | --- | --- | --- | --- |
|  | 0’ | 30’ |  | 0’ | 30’ | 120’ |  | 0’ | 30’ |
| C14:0-Cer | -0,20±0,20 | -0,66±0,51 |  | -0,25±0,29 | 0,85±0,50 | -0,21±0,15 |  | -1,32±0,94 | -1,39±1,58 |
| C16:0-Cer | 2,2±2,1 | -3,3±9,1 |  | 14,2±19,4 | -10,7±10,4 | 5,5±9,3 |  | -9,8±11,3 | 8,2±25,5 |
| C18:1-Cer | 0,03±0,07 | -0,08±0,14 |  | 0,08±0,07 | -0,24±0,19 | 0,06±0,09 |  | -0,79±0,41 | -0,84±0,61 |
| C18:0-Cer | 0,4±1,0 | 0,8±2,2 |  | 1,7±2,1 | -2,5±2,0 | 0,7±2,1 |  | -4,1±3,1 | -5,1±4,8 |
| C20:0-Cer | -1,7±1,4 | 0,5±3,5 |  | 1,1±2,1 | -2,9±2,1 | -1,4±3,5 |  | 3,4±3,4 | -2,4±4,4 |
| C22:0-Cer | -16,8±6,2 | 17,0±19,3 |  | -6,4±14,8 | -18,0±21,0 | 2,2±13,4 |  | -6,0±15,7 | -11,6±35,0 |
| C24:1-Cer | -2,8±5,4 | 27,0±16,1 |  | 3,2±13,2 | -14,9±16,4 | 10,3±20,4 |  | 3,6±19,4 | 11,1±33,9 |
| C24:0-Cer | 33,7±41,5 | 80,0±38,1 |  | 23,2±78,8 | 40,1±85,7 | 110,6±23,2 |  | -27,5±29,5 | -22,5±79,4 |
| Total | 15,1±44,3 | 121,3±63,4 |  | 36,9±58,0 | -9,2±100,0 | 128,4±49,2 |  | -42,8±61,6 | -26,3±114,1 |

The results (nmol/min/leg) are means ± SEM. Subjects performed three consecutive periods of exercise separated by 30min of rest. First subjects exercised with one leg for 30min at 25% of maximal workload (Wmax), then with the other leg for 120min at 55% of Wmax, and finally again with the first leg for 30min at 85% of Wmax. Blood samples were taken from the radial artery as well as from the femoral vein of the resting leg at indicated time points (n=10). Uptake and release of sphingolipids over the leg were calculated from arterial and femoral venous differences multiplied by plasma flow, according to the Fick principle.
